# A new, deep learning–based method for the analysis of autopsy kidney samples used to study sex differences in glomerular density and size in a forensic population

**DOI:** 10.1007/s00414-023-03153-4

**Published:** 2024-01-05

**Authors:** Valérie Vilmont, Nadine Ngatchou, Ghislaine Lioux, Sabrina Kalucki, Wendy Brito, Michel Burnier, Samuel Rotman, Christelle Lardi, Menno Pruijm

**Affiliations:** 1 Service of Nephrology and Hypertension, Lausanne University Hospital and University of Lausanne, Rue du Bugnon 17, 1011 Lausanne, Switzerland; 2 Indica Labs, Albuquerque, NM USA; 3 Service of Clinical Pathology, Lausanne University Hospital and University of Lausanne, Lausanne, Switzerland; 4 University Center of Legal Medicine Geneva, Geneva University Hospitals and University of Geneva, Geneva, Switzerland

**Keywords:** Glomerular density, Nephron number, Sex, Kidney autopsy, Artificial intelligence, Forensic medicine, Histology

## Abstract

Artificial intelligence (AI) is increasingly used in forensic anthropology and genetics to identify the victim and the cause of death. The large autopsy samples from persons with traumatic causes of death but without comorbidities also offer possibilities to analyze normal histology with AI. We propose a new deep learning–based method to rapidly count glomerular number and measure glomerular density (GD) and volume in post-mortem kidney samples obtained in a forensic population. We assessed whether this new method detects glomerular differences between men and women without known kidney disease. Autopsies performed between 2009 and 2015 were analyzed if subjects were aged ≥ 18 years and had no known kidney disease, diabetes mellitus, or hypertension. A large biopsy was taken from each kidney, stained with hematoxylin and eosin, and scanned. An in-house developed deep learning–based algorithm counted the glomerular density (GD), number, and size. Out of 1165 forensic autopsies, 86 met all inclusion criteria (54 men). Mean (± SD) age was 43.5 ± 14.6; 786 ± 277 glomeruli were analyzed per individual. There was no significant difference in GD between men and women (2.18 ± 0.49 vs. 2.30 ± 0.57 glomeruli/mm^2^, *p* = 0.71); glomerular diameter, area, and volume also did not differ. GD correlated inversely with age, kidney weight, and glomerular area. Glomerular area and volume increased significantly with age. In this study, there were no sex differences in glomerular density or size. Considering the size of the kidney samples, the use of the presented deep learning method can help to analyze large renal autopsy biopsies and opens perspectives for the histological study of other organs.

**Supplementary Information:**

The online version contains supplementary material available at 10.1007/s00414-023-03153-4.

## Introduction

The use of artificial intelligence (AI) in forensic medicine is rapidly increasing. Forensic anthropology and forensic genetics are the main areas in which AI is applied to determine the identity of the victim and/or the cause of death [1]. To the best of our knowledge, AI has never been used to study histological slides from kidneys in a forensic population. In this study, we present a new deep learning–based method to analyze histological characteristics of kidney slides taken routinely as part of forensic autopsies.

In the field of nephrology, autopsies have historically played an important role in the assessment of the average number of glomeruli and nephrons per kidney and per individual.

As such, autopsy studies (essentially performed during the last century) have shown that the number of glomeruli (and thus nephrons) varies largely between individuals, and ranges between 100,000 and over 1 million per kidney [2]. These differences in nephron number may explain why certain individuals suffer from a faster decline in kidney function throughout their life. Indeed, according to the so-called Brenner hypothesis, a reduced nephron number at birth predisposes the person to hyperfiltration in the remaining ones and can lead to chronic kidney disease (CKD) and eventually end-stage kidney disease (ESKD) [3].

Differences in glomerular density, size, or number between men and women may also explain why men are more prone to progress to ESKD than women. However, an autopsy study performed by Hoy et al. in 78 subjects found no difference in glomeruli number between men and women [4]. Denic et al. used in vivo biopsies in kidney donors and a technique based on computer tomography (CT) to assess cortical volume and calculate nephron number; they reported lower nephron numbers in women [5]. This technique may be imprecise, as it depends heavily on the nephron number and density in small in vivo biopsies that contain on average only 10–20 glomeruli [6]. Therefore, autopsy studies in this field remain the gold standard thanks to the large number of glomeruli in tissue samples. However, autopsy studies are rare, possibly because the analysis of the large samples is time-consuming and relies on manual counting of glomeruli. In this context, AI and deep learning techniques are interesting as they can considerably reduce analysis times, not only for kidneys but also for other organs. Besides, they can provide additional information that is not easily captured with the human eye, and for example, measure the glomerular perimeter, cross-sectional area, and density.

During the past decade, an increase in the use of AI in nephron-pathology of in vivo biopsies has been observed [7–10]. However, to the best of our knowledge, the use of AI in kidney autopsy samples is limited thus far [1, 11].

In this article, we present a new, fast, deep learning–based method to count glomeruli and measure glomerular cross-sectional area and density in large biopsies obtained during autopsies.

We also assessed whether this method detects differences in glomerular density, area, and volume at autopsy between men and women without known kidney disease.

## Materials and methods

### Collected data

We conducted a retrospective study, based on the medico-legal autopsies performed by the University Center of Legal Medicine of Geneva (CURML) between 2009 and 2015. The kidney biopsies were part of routine study of each case. The study protocol was approved by the Ethics Committee of the Canton de Vaud (commission cantonale d’éthique de la recherche sur l’être humain) under protocol number CER-CD 2017–01378 [12]. The study was conducted in accordance with the Declaration of Helsinki. Due to the nature of the study, an exemption for requiring informed consent was granted by the Ethics Committee of the Canton de Vaud (la commission cantonale d’éthique de la recherche sur l’être humain, CER-VD), in line with local guidelines and regulations, and the regulation on Clinical Trials in the Human Research Act article 9c and 34.

We included the autopsies of adults aged > 18 years, whose death was sudden, violent, or unexpected. We included ethnicities represented by at least five subjects. Patients were excluded in case of previously documented kidney disease or in case of documented comorbidities that may affect kidney function (diabetes mellitus or arterial hypertension). Clinical information of the subjects was obtained from numerical files in the Geneva university hospital, or by contacting the family doctors. Consumption of drugs that may induce kidney damage such as cocaine, heroin, or amphetamines (either known consumption or detection through toxicologic analysis performed at the autopsy) was also a reason to exclude the subjects. Moreover, the discovery of kidney disease at autopsy was a reason to exclude patients. To this purpose, the selection algorithm followed the KDIGO guidelines (www.kdigo.org) that define kidney disease as the presence of functional or structural (macroscopic or microscopic) abnormalities of the kidneys. Functional information (creatinine values or albuminuria at autopsy) was not available, whereas structural information was available in all due to the nature of the study. According to KDIGO, macroscopic abnormalities include polycystic or dysplastic kidneys, cortical scarring (trauma), renal masses, kidney stones, unilateral kidney, systemic atherosclerotic disease, and renal artery stenosis. Microscopic abnormalities include the presence of glomerular diseases or interstitial diseases. Patients with either macroscopic or microscopic abnormalities were excluded in our study. Severely mutilated bodies were also excluded, as well as moderate-to-severe putrefied corpses, trauma kidneys, or post-mortem interval of more than 72 h between death/death discovery and autopsy. Finally, kidney blood depletion or congestion according to the appreciation of the pathologist were also exclusion criteria, as the amount of intra-renal blood may influence glomerular density and size.

### Sampling procedure

During each autopsy, a sample of both kidneys was taken according to the local protocol for routine analyses (size of about 25 mm × 20 mm × 3 mm each). In brief, the kidney capsula was removed first, then, the kidney was sectioned longitudinally through the hilum. After excision, each kidney was weighed using a calibrated scale (SB16001, Mettler Toledo, Greifensee, Switzerland) and a large fragment was taken by the attending physician. Every block contained cortex and medulla. The sample was placed in a standard histology cassette measuring 30 × 25 × 5 mm. The material was fixed with 4% buffered formalin, then sectioned at 5 μm (thickness) using the microtome present in the histology lab (MICROM HM 340 E in older cases and LEICA RM2235 since 2012). All slides were stained with hematoxylin and eosin (H&E).

### Glomerular counting using deep learning

Scanned slides of H&E-stained renal biopsies were analyzed using HALO 3.1 software (IndicaLabs, Corrales, NM, USA). Images of slides, which were out of focus (blurred), were excluded from the analysis. For assisted artificial intelligence counting, the HALO AI DenseNet Neural Network was used with a 2- µm/px resolution and 40- µm^2^ minimum object size. Before analyzing the slides, the HALO program was trained as follows. In a first step, the program was trained to differentiate between the background and kidney tissue on a slide. In a second step, given that biopsy size can vary and result into diverse cortex vs. medulla ratio, the algorithm divided renal tissue into cortex and the medulla. Hereafter, the program was trained to count the number of glomeruli in the annotated cortical area; the surface of the total cortical area was also measured and used to calculate glomerular density (GD). In a last step, the algorithm was used to categorize glomeruli and measure their size (glomerular cross-sectional area and perimeter). Training was performed on ten scanned biopsies of varying staining intensity (very low, low, high, very high H&E intensity) up to a total of 42,000 iterations for medulla segmentation and 6700 for glomerulus segmentation. A nephrologist (MP) and pathologist (SR) with extensive experience in kidney pathology assessed the accuracy of the recognition of glomeruli, the distinction between cortex and medulla, and assessment of glomerular borders by the software during the training phase. After training, the segmentation algorithms were applied to all scanned slides of varying intensity. Glomerular count data were obtained as csv. files, converted to Excel files, and cleaned to exclude objects with an area of less than 10,000 µm^2^ identified as glomeruli. Glomerular count, glomerular cross-sectional area, glomerular perimeter, glomerular volume, and density obtained from the two kidneys were reported for each patient.

### Statistical analysis

GD was defined as the number of glomeruli per surface area of cortex (expressed in mm^2^). The mean GD of each individual was defined as the mean GD of both kidneys. Total glomerular number of each individual was the sum of glomeruli counted in the slides from the right and left kidney, whereas the mean glomerular count was the average of the number of glomeruli counted on the right- and left-sided slides. As there are no previous studies on the use of AI to count glomeruli in autopsy samples, no formal power calculation was performed. Instead, we aimed at including a similar number of cases as previous studies that used the dissector/fractionator method to manually count glomeruli and assess their volume and density [4]. Glomerular volume was calculated with the Weibel-Gomez formula (1.382 × (mean area of non-sclerotic glomeruli (NSG))^1.5^)/1.01). The volumetric glomerular density (VGD) was calculated with the following equation: 1/1.382 × $\sqrt[2]{(}$(total number of NSG/ surface area of cortex)^3^/(total area of NSG/ surface area of cortex)) [13].

Data were checked for normality using skewness and Shapiro–Wilk tests. Chi-squared tests were performed to compare categorical data between men and women (sex, smoker status, alcoholic status). Continuous clinical data (including glomerular count, area, perimeter, volume, and density) were compared with Student’s *t* test for normally distributed date and with Wilcoxon Mann–Whitney and rank sum tests for non-normally distributed data. Pearson’s correlation tests were used to assess relationship between continuous variables such as patient’s height/weight/kidney’s length, width, and glomerular data (glomerular count, area, perimeter, density, and volume). A linear regression model was applied to describe the relationship between glomerular density (outcome variable) and clinical parameters. In case the conditions of linear regression were not satisfied, a log transformation was performed. All analyses were performed with STATA 16. (StataCorp LLC). A *p*-value < 0.05 was considered as statistically significant.

## Results

### Characteristics of the study population

**Fig. 1:**
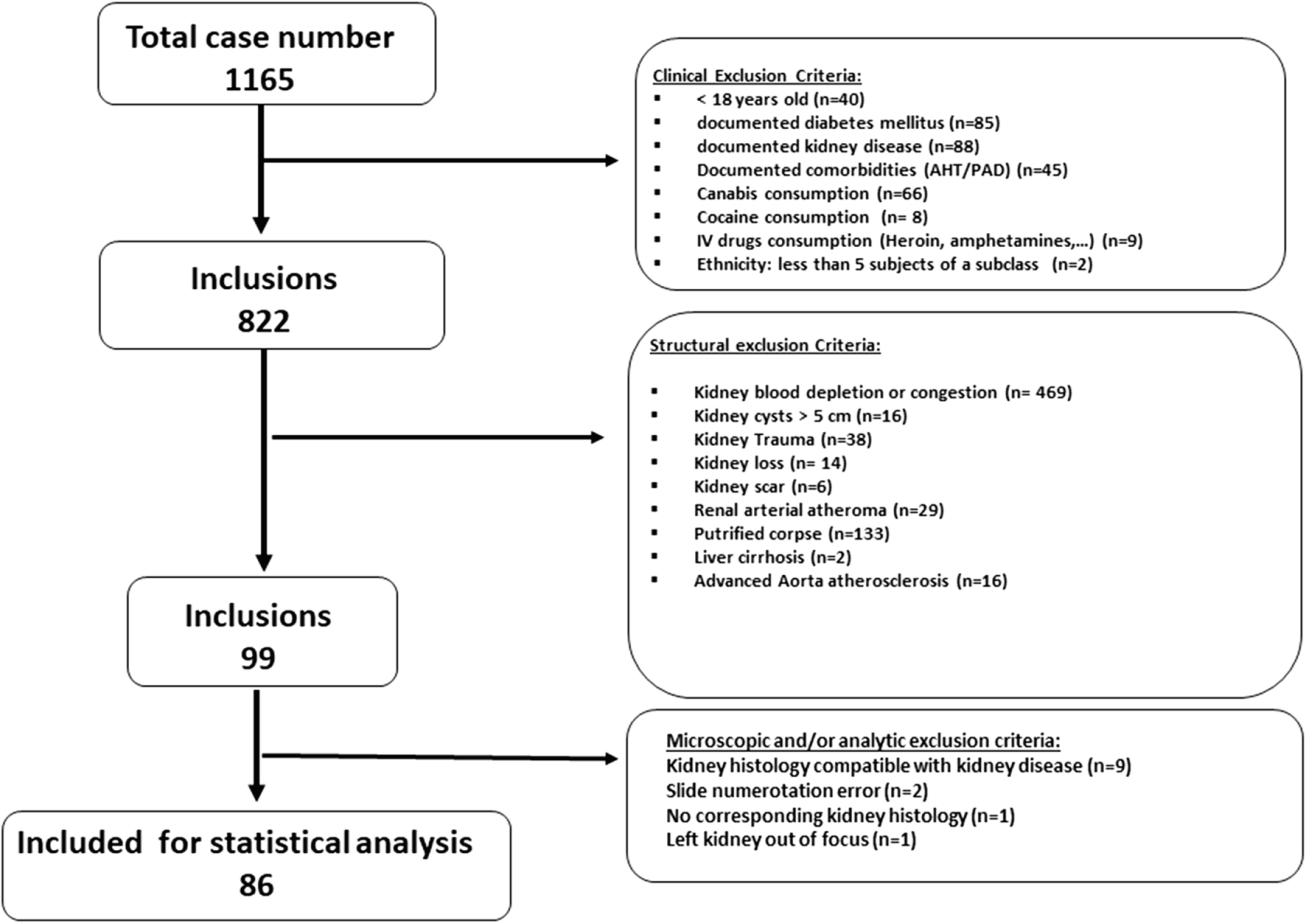
Inclusion diagram and subject selection

Out of the 1165 forensic autopsies performed by the CURML in Geneva between 2009 and 2015, 1079 were excluded for reasons outlined in the methods section, leaving 86 for this study (Fig. 1 shows the inclusion diagram and selection of subjects).

In total, 54 subjects (63%) were male. Our population was relatively young, with a mean age of 43.5 ± 14.6 years; female subjects were older than male subjects (49.0 ± 12.1 vs. 40.2 ± 15.2, *p* = 0.0062), but BMI (body mass index) was similar (see Table 1 for further details). Main causes of death were asphyxiation (16.7%), polytrauma (15.5%), drowning (10.7%), gunshots (9.5%), and intoxication (5.95%). Natural causes represented only a minority (5.95%). Causes of death were similar among men and women (see Supplementary Table S1 for more details).

**Table 1:** Clinical and biological characteristics of study population

	Male	Female
*N* (%)	54 (63)	32 (37)
Age (years, mean ± SD) (min, max)	40.2 ± 15.2 (18; 76)	49.0 ± 12.1 (20; 72)
Ethnicity, *n* (%)		
Caucasian	48 (89)	31 (97)
Subjects of African descent	6 (11)	1 (3)
Height (cm)	178.4 ± 7.1 (165; 196)	164.5 ± 7.6 (150; 180)
Weight (kg) (min, max)	79.3 ± 16.7 (47; 148)	65.0 ± 14.5 (35; 100.5)
BMI (kg/m^2^)	24.0 ± 5.0	25.0 ± 5.5
BSA (m^2^)	1.97 ± 0.19	1.70 ± 0.19
Current smoker, *n* (%)	12 (22)	7 (22)
Blood alcohol detection, *n* (%)	17 (31.5)	4 (12.9)
Chronic alcohol consumption (yes)	7 (13.0)	7 (22.6)
Kidney weight (g)	161.1 ± 5.5	127.5 ± 4.6
Kidney length (cm)	11.98 ± 1	11.64 ± 0.8

*N* number; *BMI* body mass index; *BSA* body surface area

### Artificial intelligence–assisted training of glomerular count in annotated cortical region

**Fig. 2:**
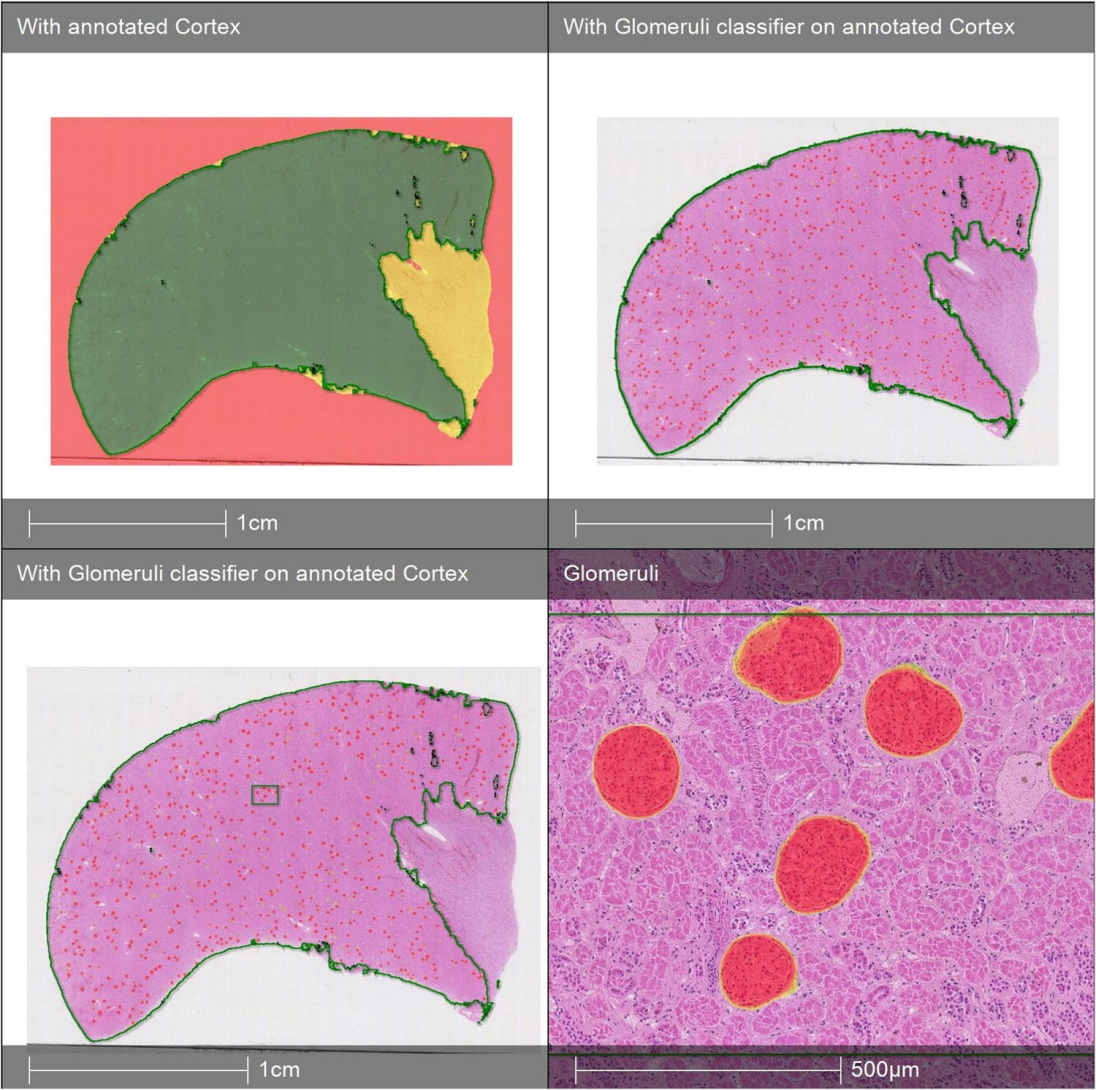
Example of the applied analysis technique to H&E-stained samples. **A** Classification of background, cortex and medulla. **B** Followed by cortex annotation and glomerular count in annotated cortex region (lower left image). Counted glomeruli in annotated cortex are shown by red dots. A close-up of the selected region is shown (lower right image)

The slides were classified into background, cortex and medulla. The “Medulla classifier” differentiated cortex from medulla. As shown in Fig. 2A, the algorithm differentiated very well between cortex and medulla, with visually a perfect match between the human eye and the algorithm. In a second step, the algorithm was trained to recognize glomeruli within the annotated cortex area (glomerular classifier). Counted glomeruli are shown as red dots (see Fig. 2 B). Again, when magnified, selected regions showed very good recognition of the typical glomerular structures (in red). Nevertheless, given that H&E staining intensity varied among slides, the algorithm had to be trained on images of different staining intensities. (see Fig. S1 in supplements). After training, a visual control of all biopsies was performed, with excellent recognition of cortex and medulla, and subjectively very good recognition of glomeruli. This was done under the direct supervision of the pathologist (SR). A detailed count was performed on one biopsy by two independent observers (MP and WB), and their results were compared with the results of the algorithm. Out of the 682 glomeruli counted by observer 1 in the biopsy, only one was erroneously counted as a glomerulus by the algorithm (false positives: 0.15%). No intact (non-sclerotic) glomeruli were missed (false negatives: 0%). Observer 2 counted 686 glomeruli; according to observer 2, two glomeruli were erroneously counted (false positive rate: 0.29%), whereas four were missed (false negative rate: 0.58%).

### Kidney characteristics, glomerular density (GD), and size

The total number of counted glomeruli by the algorithm was 67,621. A mean of 786 ± 277 glomeruli were analyzed per individual (mean ± SD). The mean glomerular count per kidney of the entire study population was 393 ± 139 (min 166, max 786). There was no significant difference in glomeruli count between men and women (*p* = 0.64), nor were there any significant differences in glomerular area, perimeter or volume between men and women (Table 2)*.*

**Table 2:** Histological characteristics

	Men 54	Women 32	*p-*value*
Glomeruli count (n per slide)			
- Right kidney	400 ± 158	398 ± 159	0.95
- Left kidney	404 ± 170	359 ± 129	0.40
- Mean of both	402 ± 146	379 ± 126	0.64
Glomerular perimeter (µm)			
- Right kidney	580.7 ± 86.3	563.6 ± 72.0	0.15
- Left kidney	579.3 ± 77.0	576.5 ± 61.8	0.93
- Mean of both	580.0 ± 79.2	570.1 ± 62.8	0.35
Glomerular cross-sectional area (µm^2^)			
- Right	24,895.3 ± 6693.1	23,113.8 ± 5426.1	0.1
- Left	24,604.2 ± 6297.6	24,167.6 ± 4688.6	0.87
- Mean of both	24,749.8 ± 6342.6	23,640.7 ± 4817.4	0.38
Glomerular volume (mm^3^)			
- Right	0.0055 ± 0.0022	0.0048 ± 0.0017	0.12
- Left	0.0054 ± 0.0022	0.0052 ± 0.0015	0.87
- Mean of both	0.0054 ± 0.0021	0.0050 ± 0.0016	0.38
Glomerular density (number/mm^2^)			
- Right	2.17 ± 0.49	2.37 ± 0.66	0.3
- Left	2.21 ± 0.58	2.24 ± 0.56	0.81
- Mean of both	2.18 ± 0.49	2.30 ± 0.57	0.71

Values expressed as mean ± SD; *SD* standard deviation

^*^Student’s *t* test was applied to compare the values between men and women

**Fig. 3:**
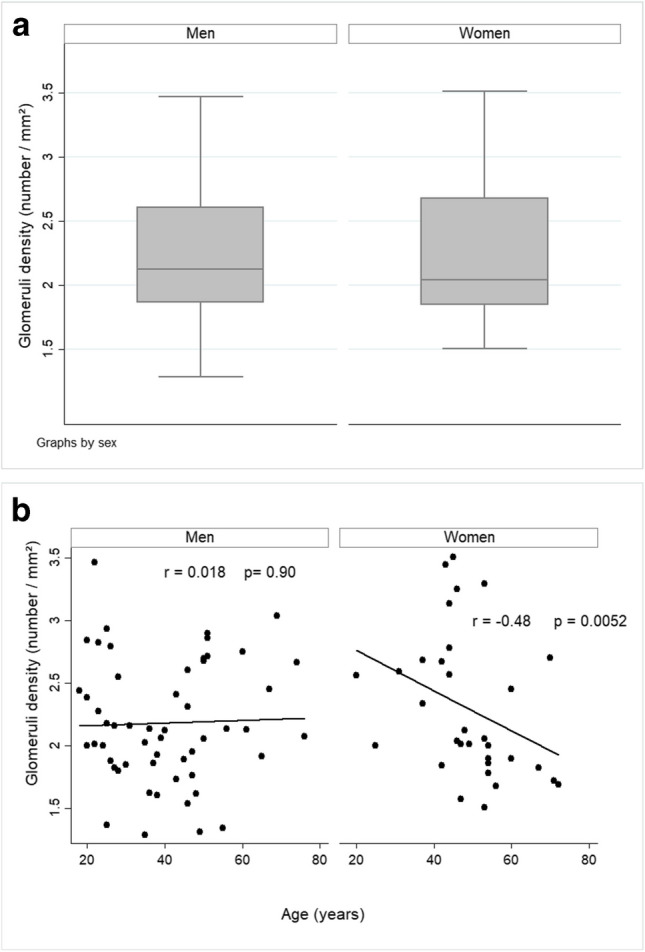
Glomeruli density by sex (**A**), correlation with age according to sex (**B**) and distribution of glomerular density in men (dotted line) and women (solid line) (**C**)

In the whole population, the mean GD (expressed as glomeruli/mm^2^) was 2.23 ± 0.52/mm^2^ (min 1.29, max 3.51), the median GD was 2.10/mm^2^ (95% confidence interval: 2.01–2.30). No significant differences in GD were found between men and women (*p* = 0.71) (Table 2 and Fig. 3 A and C). In a subgroup of 42 men and 28 women matched for age (see Table S2), GD was 2.12 ± 0.46/mm^2^ in men and 2.34 ± 0.58/mm^2^ in women (*p* = 0.26).

In our study, asphyxiation was the cause of death in 14 subjects, and drowning in 9 subjects.

When comparing the glomerular perimeter and cross-sectional area, we did not find any significant differences between the group of subjects that died because of asphyxiation or drowning and the group that died of other causes (see Supplementary Table S3).

Volumetric GD (expressed as number of glomeruli/mm^3^) also did not differ between men and women and was respectively 18.32 ± 8.18 vs. 18.57 ± 8.88 glomeruli/mm^3^, *p* = 0.89.

When comparing individuals with high versus low GD, we found that individuals with low GD had a higher kidney weight and a larger glomerular surface area (see Table 3). This was also true in analyses stratified by sex.

**Table 3:** Comparison of individuals with low vs high glomerular density

**Total population** *N* = 86			
	Low glomerular density (< median of 2.10) (mean ± SD)	High glomerular density (> median of 2.10) (mean ± SD)	*p-*value
Age (years)	45 ± 14.1	41.9 ± 15.2	0.33
Female (%)	17 (40%)	15 (35%)	0.9
BMI (kg/m^2^)	24.9 ± 5.9	24.3 ± 4.7	0.6
BSA (m^2^)	1.89 ± 0.25	1.85 ± 0.21	0.41
Kidney weight (g)	158.5 ± 43.8	138.7 ± 30.4	0.02
Glomerular area (µm^2^)	27,310 ± 883	21,364 ± 626	< 0.001
**Men** (*N* = 54)			
	Low glomerular density (< median of 2.13) *N* = 26 (mean ± SD)	High glomerular density (> median of 2.13) *N* = 28 (mean ± SD)	
Age (years)	39.7 ± 2.6	40.7 ± 3.2	0.8
BMI (kg/m^2^)	25.5 ± 1.2	24.4 ± 0.9	0.39
BSA (m^2^)	2.01 ± 0.04	1.92 ± 0.04	0.07
Kidney weight (g)	175.2 ± 8.8	148.6 ± 5.8	0.01
Glomerular area (µm^2^)	27,924 ± 1260	21,803 ± 888	0.0002
**Women** (*N* = 32)			
	Low glomerular density (< median of 2.04) *N* = 17 (mean ± SD)	High glomerular density (> median of 2.04) *N* = 15 (mean ± SD)	*p-*value
Age (years)	53.2 ± 2.7	44.3 ± 3.0	0.03
BMI (kg/m^2^)	23.9 ± 1.3	24.1 ± 1.2	0.95
BSA (m^2^)	1.70 ± 0.05	1.71 ± 0.04	0.92
Kidney weight (g)	133.8 ± 7.2	120.3 ± 5.2	0.19
Glomerular area (µm^2^)	26,372 ± 1139	20,545 ± 677	0.0003

Values are expressed as mean ± SD; *SD* standard deviation, *BMI* body mass index, *BSA* body surface area

### Factors associated with glomeruli density and size

There was no correlation between GD and age when analyzing the whole group, neither in univariate (ANOVA: *F* = 0.09, *p* = 0.91), nor in multivariate regression analysis (Table 4). However, the effect of age differed according to sex; there was a significant negative correlation between GD and age in women (Spearman’s *r* =  − 0.48, *p* = 0.005), but not in men (*r* = 0.018, *p* = 0.90). This is graphically illustrated in Fig. 3 B. Among women, individuals with high GD were significantly younger than in the low GD group (44.3 vs. 52.3 years, *p* = 0.03).

**Table 4:** Multivariable regression analysis showing the association between glomerular density (outcome variable) and clinical and histological variables

Glomerular density	Univariate regression analysis		Multivariate regression analysis	
	Unadjusted *β* (95%CI)	*p*	Fully adjusted *β* (95%CI)	*p*
Sex (women vs men)	0.11 (− 0.12; 0.35)	0.34		
Age (per year)	− 0.002 (− 0.01; 0.0056)	0.57		
BMI (per kg/m^2^)	− 0.008 (− 0.03; 0.013)	0.45		
Body Height (per cm)	− 0.012 (− 0.023; − 0.00009)	0.048	− 0.013 (− 0.027; 0.0007)	0.06
Kidney weight (per g)	− 0.005 (− 0.008; − .0.0026)	0.0002	− 0.002 (− 0.005; 0.0015)	0.27
Glomerular surface area (per µm^2^)	− 0.00005 (− 0.00006; − 0.00003)	< 0.0001	− 0.00004 (− 0.00006; − 0.00002)	< 0.0001

The fully adjusted model included all the variables that were significantly associated with glomerular density in univariate analysis. *BMI* body mass Index

**Fig. 4:**
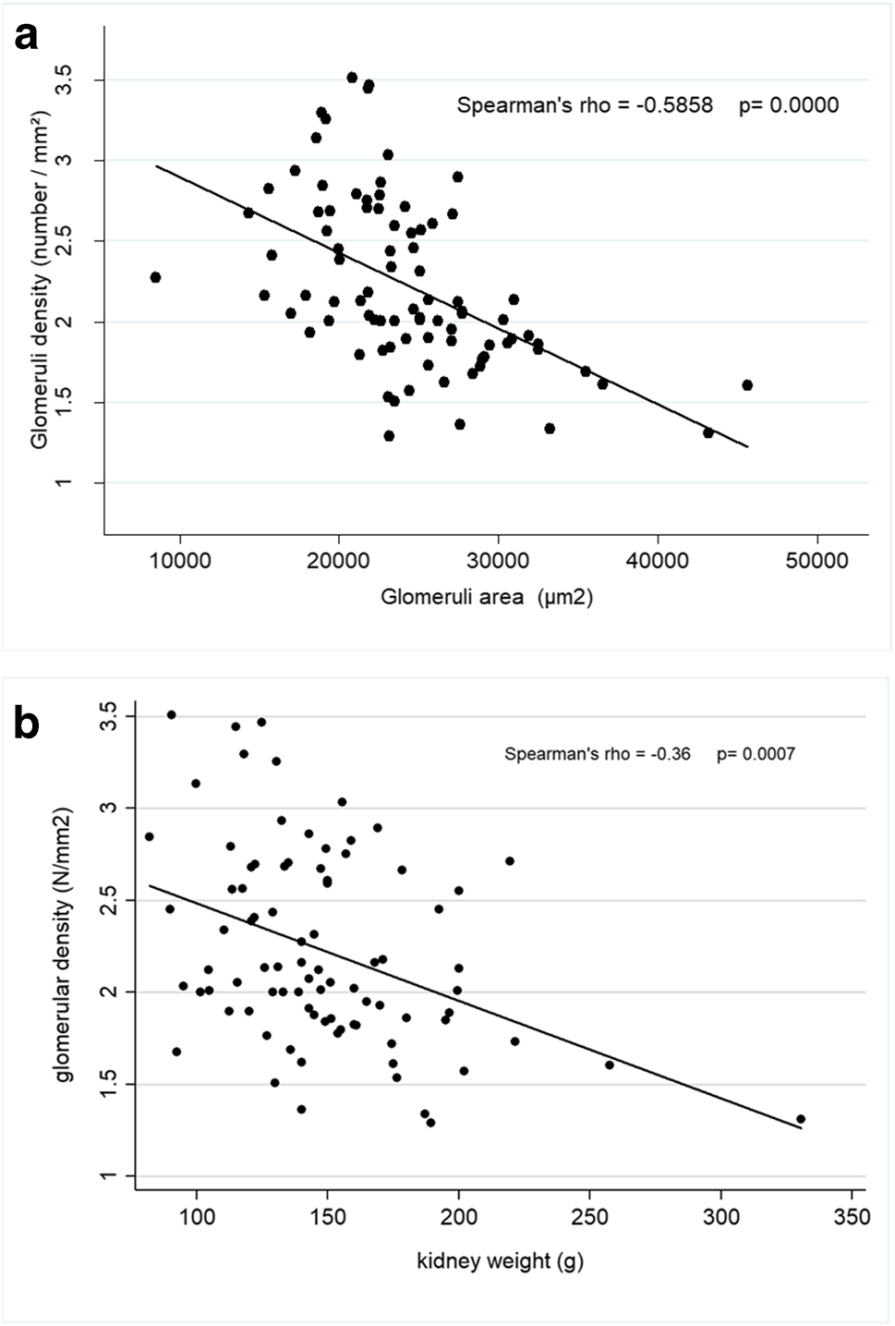
Correlation between glomerular density, glomerular area and kidney weight. **A** correlation between glomerular density and glomerular area. **B** correlation between glomerular density and kidney weight

Besides, we found a negative correlation between the GD and the glomeruli surface area (Spearman’s rho =  − 0.59, *p* < 0.0001), meaning that as GD decreased, the glomeruli area (size) increased (Fig. 4A).

GD was also inversely correlated with kidney weight (see Fig. 4B). This association was still present when two outliers were removed from the analysis.

In univariate analysis, GD was associated with body height, kidney weight, and glomerular surface area. In multivariable regression analysis, GD was only associated with glomerular surface area (Table 4). This association was present in both women and men when analyses were stratified by sex (Table S4 A and B).

In a group of 20 subjects with information on kidney length, GD did not vary significantly with kidney length. (Spearman − 0,09, *p* = 0.71). In the same group of 20 patients, we found a positive correlation between glomerular cross-sectional surface and kidney length (Spearman’s rho 0.48, *p* = 0.031). Glomerular surface was independently associated with age (older age) and kidney weight.

According to Weibel-Gomez formula, the mean glomerular volume (GV) was 0.0053 ± 0.0019 mm^3^. We found no difference in glomerular volume between men and women. The glomerular volume (GV) correlated (just like the glomerular surface area) negatively with glomerular density (Spearman’s *r* = 0.59 and *p* < 0.0001). Besides, GV correlated positively with kidney weight, BSA, age, and BMI.

When VGD instead of GD was taken as outcome variable, an association was found with kidney weight and glomerular surface area in univariate analysis, but not with body height. In multivariate analysis, VGD was only associated with glomerular surface area (see Table S5 and S6).

## Discussion

The main findings of this autopsy study are as follows: (1) the use of trained artificial intelligence to analyze large samples of post-mortem kidney tissue is accurate and time-saving, (2) no significant differences were detected in glomerular density or size between men and women, and (3) glomerular density correlated inversely with glomerular size and kidney weight.

In total, 67,621 glomeruli were counted by the HALO AI Dense Net Neural Network, demonstrating a real advantage of using such technology in the counting of large numbers of glomeruli in human kidney tissue. Once trained, mean analysis time per sample was ~ 15 min; this achievement would be impossible for the human eye, considering that over 750 glomeruli were counted and measured per subject. However, training of the network was challenging, as autopsy samples were stained with H&E. This technique leads to a rather homogenous color of the tissue and no clear borders between glomeruli and tubuli as compared to PAS (periodic acid Shiff), or methenamine–silver staining for example. We also had to train the program at different staining intensities.

We recognize that further improvements are possible. For example, our program identified correctly healthy glomeruli, but did not differentiate between partly sclerotic or intact glomeruli, whereas completely sclerotic glomeruli were not counted. This was because the program had been trained to recognize intact, non-sclerotic glomeruli. The number of partly or completely sclerotic glomeruli was relatively low in our study (< 5% at visual control), so we believe that this had no major impact on our results, but future improvements should overcome this limitation.

In support of the adequacy of our technique, the mean GD in our study was 2.23 ± 0.52 per mm^2^. This is similar to the density reported by Rule et al., which was 2.3 ± 0.8 /mm^2^ on biopsies performed in living kidney donors [14]. In contrast, Tsuboi et al. reported a substantially higher GD of 3.1 ± 1.0/mm^2^ in living donors [15]. Our study population, like the one in the study by Rule et al., included mainly Caucasians subjects, whereas the study by Tsuboi et al. included exclusively Asian subjects. It is therefore possible that GD is higher in Asians. This needs to be confirmed in dedicated studies.

We did not find a statistical difference in the GD between men and women. Knowing that women have a smaller absolute and relative kidney size than men, this finding suggests that the number of glomeruli is lower in women than in men, in accordance with some previous studies [4, 12]. However, GD is not equal to nephron number, but is used as a proxy. Besides, we had no information on cortical volume. This could have permitted us to estimate the total nephron number per kidney. Larger studies that also analyze cortical volume are necessary to reach definite conclusions. This study should therefore be considered as a pilot study that shows the potential of the AI Halo 3.1 software in the analysis of large autopsy samples and demonstrates the utility of AI for the study of normal anatomy and histology in forensic medicine.

GD was negatively and strongly correlated with glomerular cross-sectional surface and volume. A similar result was previously found by Rule et al. [14]. This can be seen as an argument in favor of Brenner’s hypothesis that states that as glomerular count decreases, glomerular hypertrophy (glomerulomegaly) and hyperfiltration develop in the remaining ones [4]. Once more, this finding should be interpreted with caution. In case of glomerulomegaly, tubuli also increase in size, and as tubuli represent 95% of nephron volume, this leads automatically to a lower GD. Individuals with low GD had not only a higher glomerular surface but also a higher kidney weight. This association was significant in univariate, but not in multivariate regression analysis. Hence, our data do not contradict the general accepted paradigm that kidney size is positively associated with nephron number.

A younger age was associated with a higher GD in women, but not in men. This may support the idea that the number of glomeruli decreases with aging, at least in women. Denic et al. also reported a reduction in glomeruli number with increasing age [5]. Hoy et al. found a strong correlation between sclerosed glomeruli and increasing age, but there was no difference between men and women in their study [4]. Why GD did not decrease with age in men in our study is not clear. This may be linked to their lower mean age and smaller variability in age.

This study has several limitations. First of all, we had no information on creatinine values or albuminuria, and solely relied on numeric files and histological data to exclude subjects with kidney disease. Secondly, renal and cortex volumes were not measured, and therefore the exact number of glomeruli per kidney could not be calculated. Thirdly, the study included mainly Caucasians, and our results can therefore not be generalized to other ethnicities. Finally, the number of subjects was low, and no formal power calculation was performed as this was a pilot study. The described results and associations should therefore be interpreted with caution and confirmed in larger studies.

The principal strength of our study is the use of artificial intelligence in analyzing large autopsy samples (digital histology analysis), which allowed an important gain of time and resources. The study also shows how forensic pathology morphometrical studies can benefit from AI-based techniques to analyze human tissues. Artificial intelligence can provide detailed information that cannot be easily obtained with manual techniques; for the kidneys, information on glomerular density, perimeter, volume, and surface were easily extracted.

## Conclusion

In conclusion, this study adds to the growing knowledge that “digital pathology” methods can be of great help in forensic research, particularly for the analysis of large kidney samples obtained at autopsy. We did not find differences in glomerular density and number between men and women, but future AI-based autopsy studies that include measurement of cortical volume will be able to confirm or reject this statement.

### Supplementary Information

Below is the link to the electronic supplementary material.

Supplementary file1 (DOCX 795 KB)

## Data Availability

On request to the principal investigator (MP).
