# Supplementary material for: A new, deep learning–based method for the analysis of autopsy kidney samples used to study sex differences in glomerular density and size in a forensic population
Source: Int J Legal Med. 2024 Jan 5;138(3):873–82. doi: 10.1007/s00414-023-03153-4 (PMC11003899; doi:10.1007/s00414-023-03153-4)
Supplement: Supplementary file 1 — Supplementary file1 (DOCX 795 KB) [file 414_2023_3153_MOESM1_ESM.docx]

**SUPPLEMENTS**

**Figure S1:** Recognition of glomeruli by HALO 3.1 software in slides of different H&E staining intensity. A) Very-low and very-high intensity images. B) Low and high intensity images.


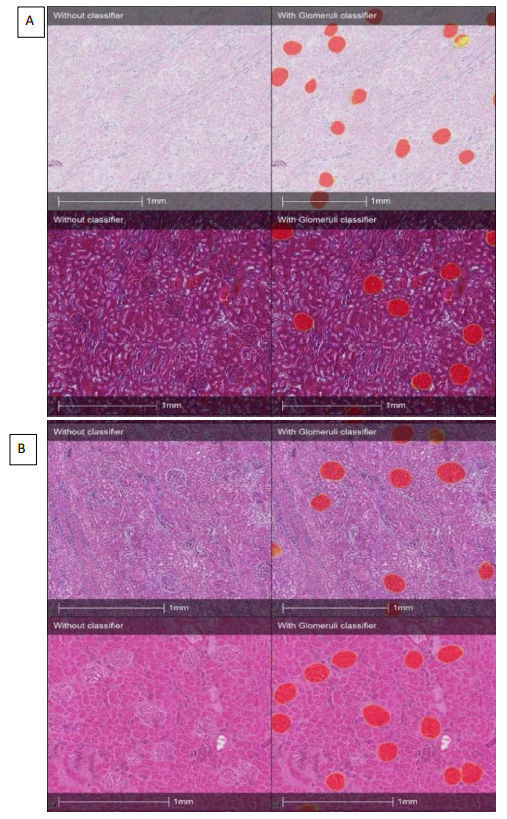


**Table S1 :** Causes of death.

| **Cause of death** | **Total population**  Number (%) | **Men**  Number (%) | **Women**  Number (%) |
| --- | --- | --- | --- |
| Natural unknown | 5 (5.95) | 4 (7.55) | 1 (3.22) |
| Cardiac | 3 (3.57) | 3 (5.66) |  |
| Lung | 2 (2.38) | 1 (1.89) | 1 (3.22) |
| Vascular | 1 (1.19) | 1 (1.89) | - |
| Other natural | 4 (4.76) | - | 4 (12.90) |
| Intoxication | 5 (5.95) | 1 (1.89) | 5 (16.13) |
| Asphyxiation | 14 (16.67) | 11 (20.75) | 3 (9.68) |
| Polytrauma | 13 (15.48) | 9 (16.98) | 4 (12.9) |
| Drowning | 9 (10.71) | 4 (7.55) | 5 (16.12) |
| White weapon | 5 (5.95) | 4 (7.55) | 1 (3.22) |
| Gunshot | 8 (9.52) | 6 (11.32) | 2 (6.45) |
| Other unnatural | 15 (17.86) | 10 (18.87) | 5 (16.12) |
| **Total** | 84 * | 53 | 31 |

*Cause of death unknown in 2 subjects.

**Table S2**: glomerular size according to cause of death (asphyxiation-drowning versus death due to other causes).

|  | Drowning/asphyxia  23 | Other causes of death  63 | *p-value** |
| --- | --- | --- | --- |
| Age at death (years, mean ± SD)  (min, max) | 45.5 ± 12.5  (25, 71) | 42.7 ± 15.4  (18, 76) | 0.39 |
| Female (%) | 8 (35%) | 24 (38%) | 0.98 |
| Glomerular perimeter (µm)   - Right Kidney - Left Kidney - Mean of Both | 595.9 ± 59.5  583.7 ± 60.6  589.8 ± 57.3 | 566.5 ± 86.9  576.3 ± 75.2  571.4 ± 78.2 | 0.08  0.64  0.24 |
| Glomerular cross-sectional area (µm²)   - Right - Left - Mean of both | 25693.4 ± 4889.9  24905.2 ± 4723.2  25299.3 ± 4606.5 | 23699.0 ± 6669.2  24272.6 ± 6077.1  23985.8 ± 6196.2 | 0.14  0.61  0.29 |

**Table S2**. Clinical and biological characteristics of a subgroup of the study population matched for age.

|  | Men | Women |
| --- | --- | --- |
| Number | 42 | 28 |
| Age (years, mean ± SD)  (min,max) | 45.3 ± 13.2  (26 ; 76) | 46.0 ±9.6  (20 ; 60) |
| Ethnicity, number (%)  Caucasian  Subjects of African descent | 37 (88 )  5 ( 12) | 27 (96 )  1 (4 ) |
| Height (cm) | 178.2±6.8  (165 ; 193) | 164.0 ± 7.5  (150 ; 180) |
| Weight (kg)  (min,max) | 81.3 ±17.2  (58 ; 148) | 65.4±14.2  (49 ; 100.5) |
| BMI (kg/m^2^ ) | 25.7±5.9 | 25.0±5.5 |
| Kidney weight (g) | 167.5±40.5 | 124.1.5±25.5 |
| Glomerular density (number / mm²) | 2.12±0.46 | 2.34±0.58 |
| Glomerular surface area (per µm²) | 26041±6123 | 22780±4072 |

BMI= Body mass index; BSA= Body surface area.

**Table S3:** Glomerular size according to cause of death, comparing those that died because of asphyxiation or drowning to those that died of other causes.

|  | Drowning/asphyxia  23 | Other causes of death  63 | *p-value** |
| --- | --- | --- | --- |
| Age at death (years, mean ± SD)  (min, max) | 45.5 ± 12.5  (25, 71) | 42.7 ± 15.4  (18, 76) | *0.39* |
| Female (%) | 8 (35%) | 24 (38%) | *0.98* |
| Glomerular perimeter (µm)   - Right Kidney - Left Kidney - Mean of Both | 595.9 ± 59.5  583.7 ± 60.6  589.8 ± 57.3 | 566.5 ± 86.9  576.3 ± 75.2  571.4 ± 78.2 | 0.08  0.64  0.24 |
| Glomerular cross-sectional area (µm²)   - Right - Left - Mean of both | 25693.4 ± 4889.9  24905.2 ± 4723.2  25299.3 ± 4606.5 | 23699.0 ± 6669.2  24272.6 ± 6077.1  23985.8 ± 6196.2 | 0.14  0.61  0.29 |

**Table S4A:** Multivariable regression analysis showing the association between glomerular density (outcome variable) and clinical and histological variables in men (n=54).

| Glomerular density | Univariate regression analysis | | Multivariate regression analysis | |
| --- | --- | --- | --- | --- |
|  | Unadjusted β (95%CI) | *p* | Fully adjusted β (95%CI) | *p* |
| Age (per year) | 0.001 (-0.008 ; 0.01) | 0.8 |  |  |
| BMI (per kg/m²) | -0.011 (-0.036 ; 0.014) | 0.38 |  |  |
| Body Height (per cm) | - 0.022 (-0.04 ;-0.003) | **0.024** | -0.026 (-0.045 ; - 0.006) | **0.01** |
| BSA (m²) | -0.77 (-1.48 ; -0.58) | **0.035** | 0.46 (-0.40 ; 1.31) | 0.3 |
| Kidney weight (per g) | -0.005 (-0.009 ; -.0.002) | **0.001** | -0.003 (-0.007 ; 0.001) | 0.2 |
| Glomerular surface area  (per µm²) | -0.00004 (-0.00006; -0.00002) | **<0.001** | -0.00003 (-0.00006; -0.00001) | **0.004** |

**Table S4B:** Multivariable regression analysis showing the association between glomerular density (outcome variable) and clinical and histological variables in women (n=32).

| Glomerular density | Univariate regression analysis | | Multivariate regression analysis | |
| --- | --- | --- | --- | --- |
|  | Unadjusted β (95%CI) | *p* | Fully adjusted β (95%CI) | *p* |
| Age (per year) | -0.016 (-0.033 ; 0.0008) | 0.061 |  |  |
| Body Height (per cm) | - 0.0059 (-0.035 ; 0.023) | 0.68 |  |  |
| BMI (per kg/m²) | -0.00001 (-0.044 ; 0.044) | 1.0 |  |  |
| Kidney weight (per g) | -0.007 (-0.015 ; 0.00045) | 0.064 |  |  |
| Glomerular surface area  (per µm²) | -0.00007 (-0.0001; -0.00004) | **<0.001** | -0.00006 (-0.0001; -0.00002) | **0.003** |

**Table S5:** Multivariable regression analysis showing the association between glomerular density (outcome variable) and clinical and histological variables (including glomerular volume instead of glomerular surface area).

| Glomerular density | Univariate regression analysis | | Multivariate regression analysis | |
| --- | --- | --- | --- | --- |
|  | Unadjusted β (95%CI) | *p* | Fully adjusted β (95%CI) | *p* |
| Sex (women vs men) | 0.11 (-0.12 ; 0.35) | 0.34 |  |  |
| Age (per year) | -0.002 (-0.01 ; 0.0056) | 0.57 |  |  |
| BMI (per kg/m²) | -0.008 (-0.03 ; 0.013) | 0.45 |  |  |
| Body Height (per cm) | - 0.012 (-0.023 ;-0.00009) | **0.048** | - 0.011 (-0.025 ; 0.003) | 0.12 |
| Kidney weight (per g) | -0.005 (-0.008 ; -.0.0026) | **0.0002** | -0.002 (-0.005 ; 0.0015) | 0.27 |
| Glomerular volume (per ml) | **-140.72 (-190.75 –90.69)** | **<0.001** | **-133.81 (-199.05 -68.59)** | **<0.001** |

**Table S6:** Multivariable regression analysis showing the association between volumetric glomerular density (outcome variable) and clinical and histological variables.

| Volumetric glomerular density  (VGD) | Univariate regression analysis | | Multivariate regression analysis | |
| --- | --- | --- | --- | --- |
|  | Unadjusted β (95%CI) | *p* | Fully adjusted β (95%CI) | *p* |
| Sex (women vs men) | 0.25 (-3.50 ; 4.00) | 0.90 |  |  |
| Age (per year) | -0.08 (-0.21; 0.04) | 0.18 |  |  |
| Body Height (per cm) | 0.010 (-0.18;-0.19) | 0.92 |  |  |
| BMI (per kg/m²) | - 0.08 (-0.42; 0.26) | 0.65 |  |  |
| Kidney weight (per g) | -0.066 (-0.11 ; -.0.02) | **0.004** | -0.024 (-0.073 ; 0.025) | 0.34 |
| Glomerular surface area  (per µm²) | -0.0006 (-0.0009; -0.0004) | **<0.001** | -0.0005 (-0.0087; -0.0002) | **<0.001** |
